# Supplementary material for: Oxide Membranes from Bulk Micro‐Machining of SrTiO3 Substrates
Source: Adv Sci (Weinh). 2025 May 8;12(20):2412683. doi: 10.1002/advs.202412683 (PMC12120795; doi:10.1002/advs.202412683)
Supplement: Supplementary file 1 — Supporting Information [file ADVS-12-2412683-s001.pdf]

ADVANCED SCIENCE
Open Access

Supporting Information

for *Adv. Sci.*, DOI 10.1002/advs.202412683

Oxide Membranes from Bulk Micro-Machining of $SrTiO_3$ Substrates

Nicola Manca, Alejandro E. Plaza, Leonélio Cichetto, Warner J. Venstra, Cristina Bernini, Daniele Marré and Luca Pellegrino*

Oxide Membranes from Bulk Micro-Machining of $SrTiO_3$ substrates

Nicola Manca,[1,2,*] Alejandro E. Plaza,[1,2] Leonélio Cichetto Jr,[1]
Warner J. Venstra,[3] Cristina Bernini,[1,2] Daniele Marré,[4,1] and Luca Pellegrino[1,2]

[1]*CNR-SPIN, C.so F. M. Perrone, 24, 16152 Genova, Italy*

[2]RAISE *Ecosystem, Genova, Italy*

[3]*Quantified Air BV, Langegracht 70, 2312NV Leiden, The Netherlands*

[4]*Dipartimento di Fisica, Università degli Studi di Genova, 16146 Genova, Italy*

This supplemental material contains the following:

- Section I: X-ray diffraction analysis of LSMO thin films
- Section II: Measurement protocol for the calibration of the etching rates
- Section III: Evolution of the sidewalls with the etching time: form etch pits to large openings

* nicola.manca@spin.cnr.it

Sec. I. X-RAY DIFFRACTION ANALYSIS OF LSMO THIN FILMS

X-ray diffraction was performed on a Bruker D8 discover Diffractometer equipped with a IμS High Brillance microfocus X-ray source. Figure S1 shows ϕ-scan of the (103) peak of both LSMO film and STO substrate. It confirms epitaxial relationship with cube-on-cube growth mode. Phase identification was performed by standard θ–2θ scans, as reported in Figure S2. After immersing the samples in 5 % HF water solution for 1 hour, θ–2θ scans were repeated to check whether the process affected the lattice constant of the LSMO film. No significant difference was identified.

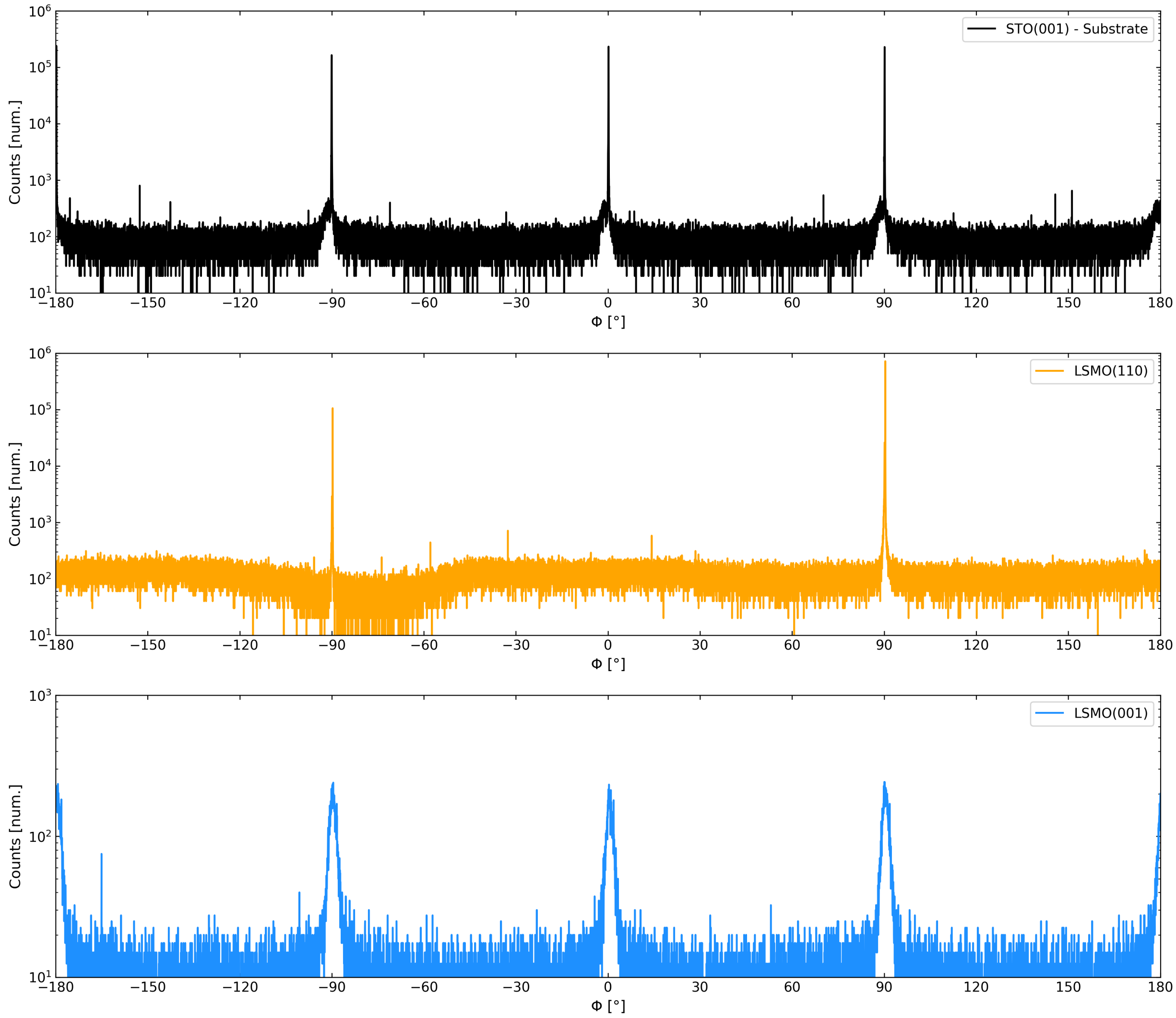

Figure S1. ϕ-scans of the STO(001) substrate, the (001) peak of a LSMO(110) films and the (103) peak of a LSMO(001) film.

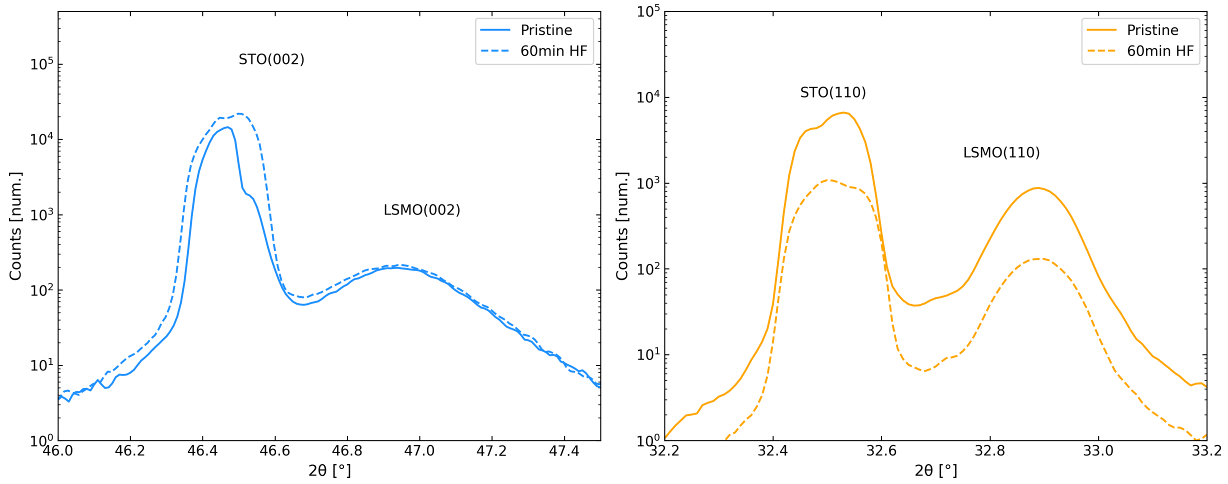

Figure S2. θ–2θ scans of the (002) peak of LSMO(001) and the (011) peak of LSMO(011) in pristine condition (continuous line) and after 1 h immersed in HF (dashed line).

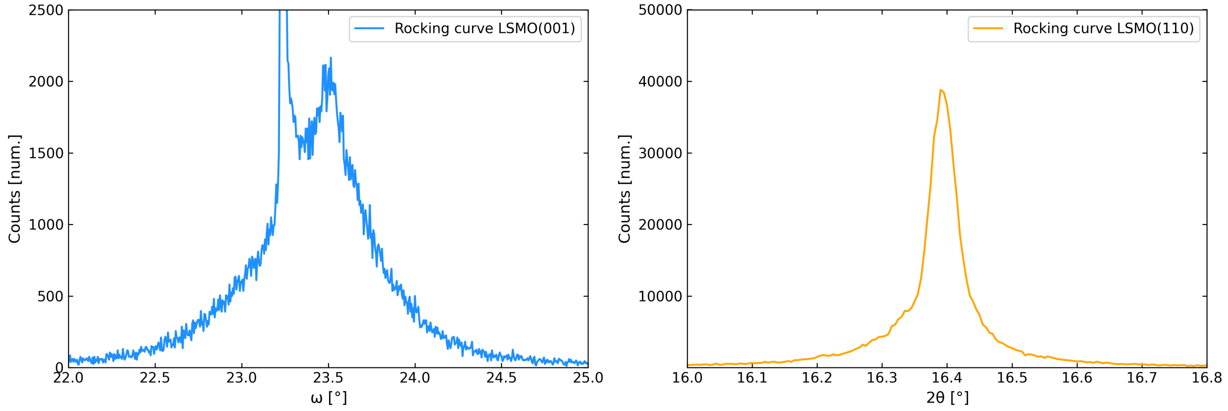

Figure S3. ω scans (rocking curve) of a LSMO(001) and LSMO(110) thin films. Full width at half maximum of the two curves is 0.5 and 0.05 degrees, respectively.

Sec. II. MEASUREMENT PROTOCOL FOR THE CALIBRATION OF THE ETCHING RATES

We employ an optical microscope (50× objective) to focus at first on the top sample surface covered by a 100 nm thick (La,Sr)MnO_3 mask and subsequently on the bottom part of the etched region. We extract the etching depth by measuring the difference of the z-value indicated on the graduated microscope knob.

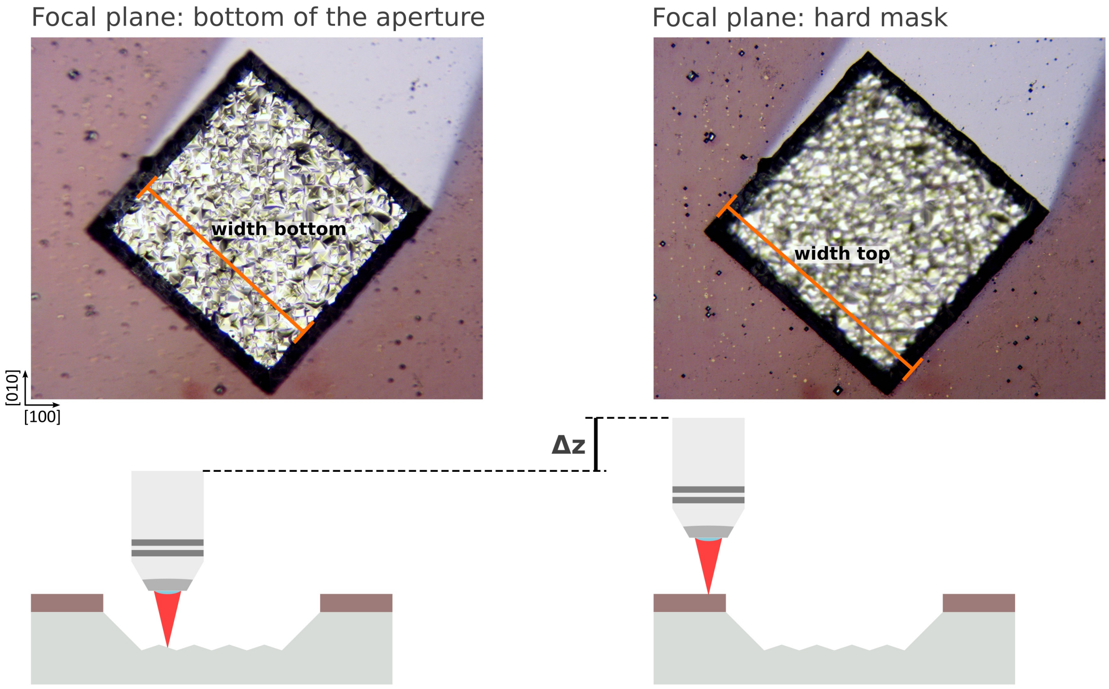

Figure S4. Example of the measurement protocol of bottom width, top width, and dept of the aperture in the STO(001) substrate due to HF etching.

Sec. III. EVOLUTION OF THE SIDEWALLS WITH THE ETCHING TIME: FORM ETCH PITS TO LARGE OPENINGS

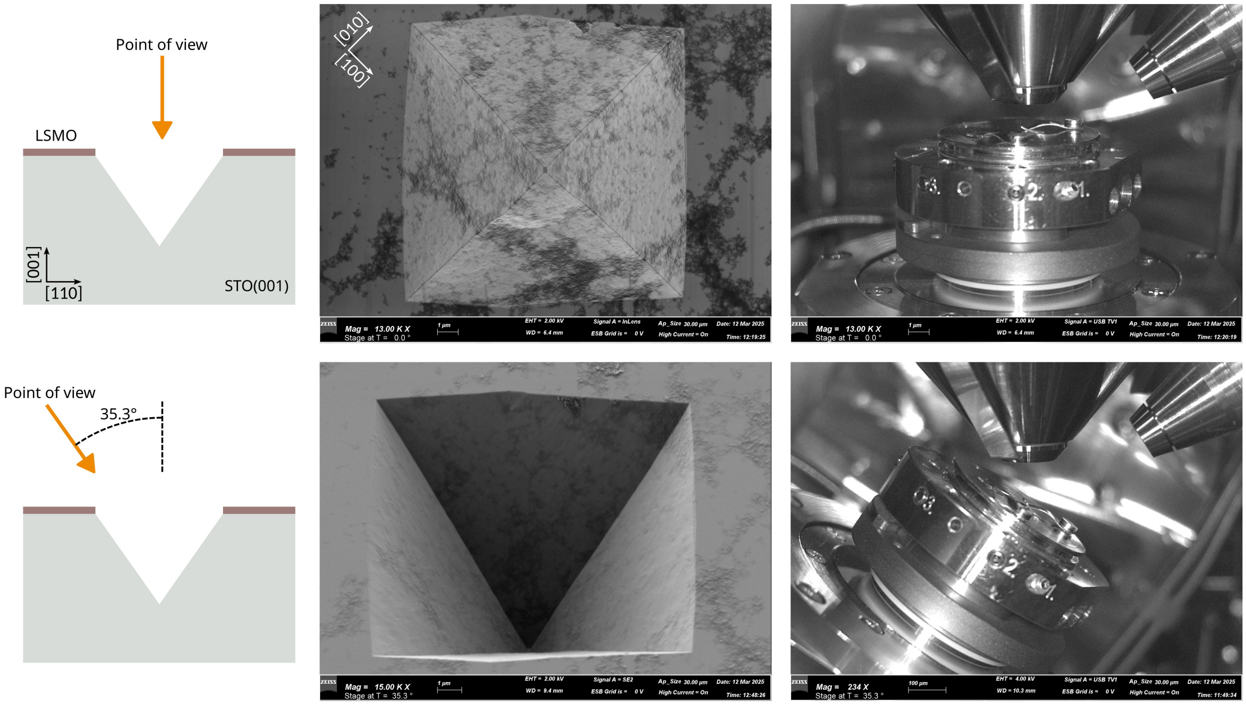

Figure S5. Measurement by scanning electron microscope of the sidewall angles of an etch pit formed on the $SrTiO_3$(001) surface after immersion in HF solution. For both the observation angles (top row 0°, bottom row 35.3°) we show a sketch of the point of view, the corresponding SEM image (top: InLens mode, bottom: SE2 mode), and the sample as positioned in the SEM chamber. The sample surface is covered by scattered contaminants likely due to photoresist and etching by-products. The free-hanging LSMO mask on the etch pit has been removed after the fabrication by sonicating the sample in ethanol bath.

The profile of the apertures' sidewalls evolves with the etching time because the defects open new accessible surfaces to the etching solution. This fact means that the angle and the smoothness of the sidewalls change with their vertical dimension. HF enters through point defects of the LSMO hard mask and forms etch pits on the substrate surface, as visible in Figure S5. The etch pits have small lateral size if compared to the aperture size employed to evaluate the etching rates and discussed in the main text (see Figure 1). Concerning in-plane directions, the sidewalls of the etch pits show a spontaneous rotation of 45° with respect to main lattice directions. These sidewalls have a

slope of ∼35° with respect to the perpendicular out-of-plane direction. This is measured by tilting the sample in a scanning electron microscope until the selected sidewalls becomes parallel to the optical path. The measurements of the in-plane sidewalls direction and their vertical angle allow us to identify the exposed sidewalls as parallel to the {111} lattice planes.

In Figure S6 we show another etch pit of ∼5 µm lateral size formed on the STO(001) surface. From a macroscopic point of view, its geometry perfectly matches with what expected from our previous analysis: flat sidewalls parallel to the {111} STO lattice planes. However, after further magnification, the sidewalls of this etch pit show signs of chemical etching, as indicated by the arrows. These signs are the starting points of new etch pits that will form on the sidewall and are likely determined by local defects in the STO crystal. The length scale of these defects is sub-micrometric.

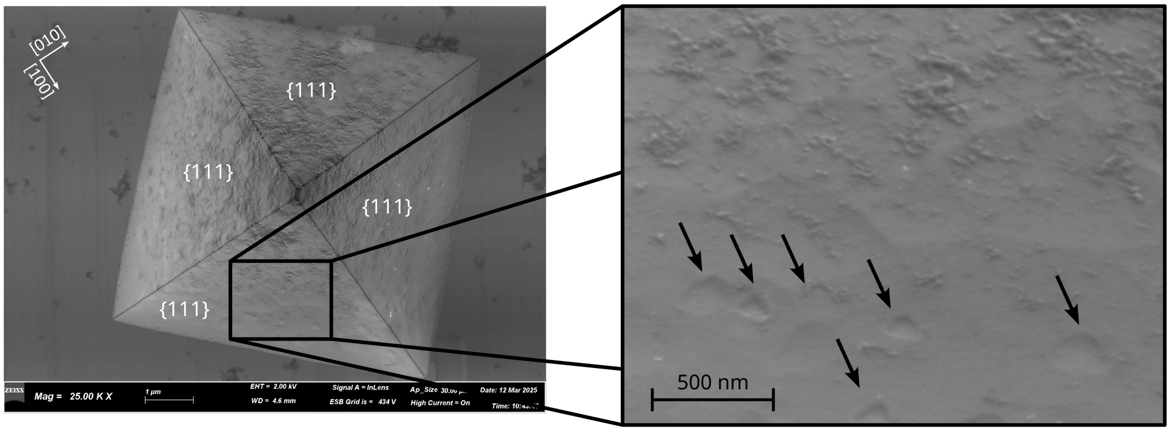

Figure S6. SEM image (InLens mode) of an etch pit as that reported in Figure S5. The zoomed image shows the initial etching of the {111} planes.

We can compare the previous results to the time evolution of the geometric parameters of the apertures as reported in Figure 1 of the main text. From the reported top/bottom width ($w_{t/b}$) and depth (d) data we calculate the slope of the sidewalls at each measurement time as $\theta = (w_t - w_b)/(2d)$. The resulting angles are reported in the scatterplots of Figure S7, where we perform this analysis for the STO(001) case (top) and the STO(110) case in the (001) in-plane direction (bottom). These angles are higher than those of vicinal {111} planes and increases over time. We visually compare the profile of the sidewalls in the central panels of Figure S7. The black dashed lines are the sidewalls as expected if

the {111} plane was fully resistant to HF, while the solid lines are the actual sidewalls at the end of the etching process. In both the cases the LSMO hard mask is visible in brown colour. The difference between the theoretical and actual sidewalls geometry shows that the etching front evolution is mediated but not bound to the HF-resistant {111} planes. Instead, it is a dynamical process where other mechanisms also play an important role, such as the formation of etch pits at the sidewalls that are visible in the SEM images. These etch pits are due to crystal defects and allow the etch front to penetrate in the in-plane direction. As a result, the sidewalls' angle changes over time, as shown in the scatter plot of Figure S7.

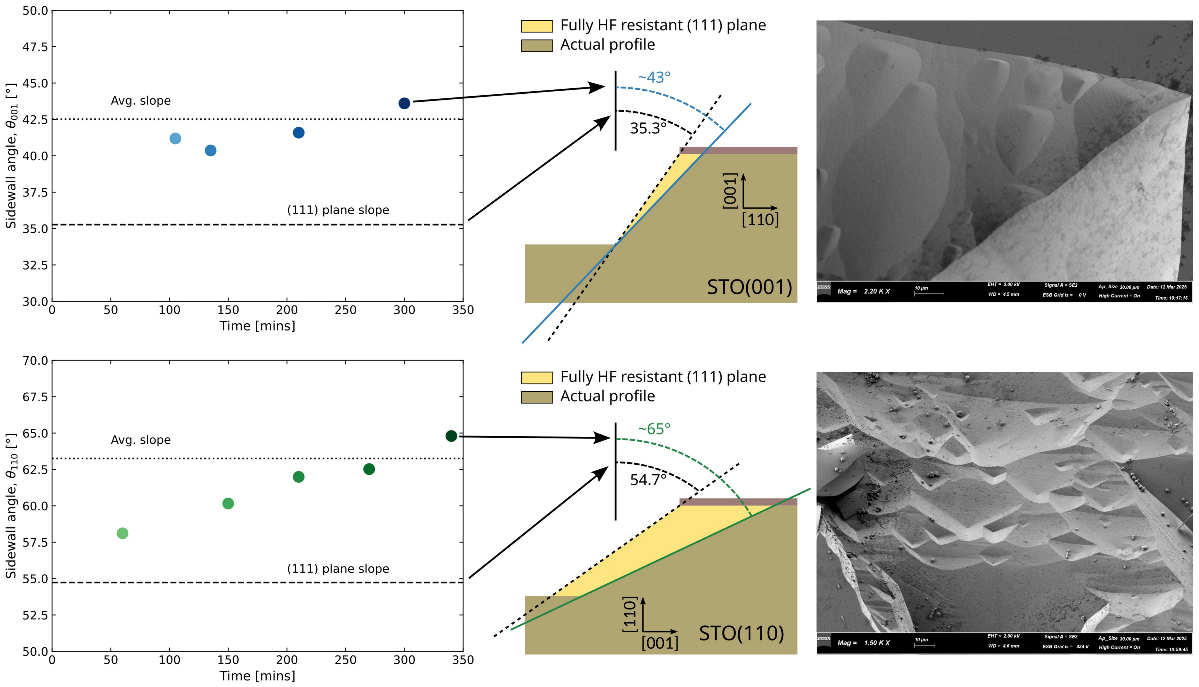

Figure S7. Time plots (left) of the sidewalls angle (dots) in comparison to 111 planes slope (dashed line) and average slope from Table 2 of the main text (dotted line). Top row corresponds to STO(001) and bottom row to STO(110) along the [001] direction. Sketches of the sidewalls (centre) at the end of the etching process compared with the slope of their vicinal (111) plane. SEM pictures (right) of the sidewalls at the end of the etching process showing the presence of etch pits determining the observed time-dependent angle of the sidewalls.

Concerning the $[1\bar{1}0]$ sidewall direction, in the first minutes of the etching process they are vertically aligned, i.e parallel to the $\{1\bar{1}0\}$ planes. This is visible in Figure 3 of Ref. 43,

showing 10 µm-deep sidewalls after 30 mins of etching time. In the deep-etching regime discussed in this work, the etch pits dynamics affects the etch front evolution resulting in a final slope of about 11°. However, it was not possible to perform a time-dependent analysis of this angle as in the other directions discussed above. This is because the presence of defects prevents a reliable evaluation of the average slope of the sidewalls as visible in Figure S8, measured after 100 µm etching depth.

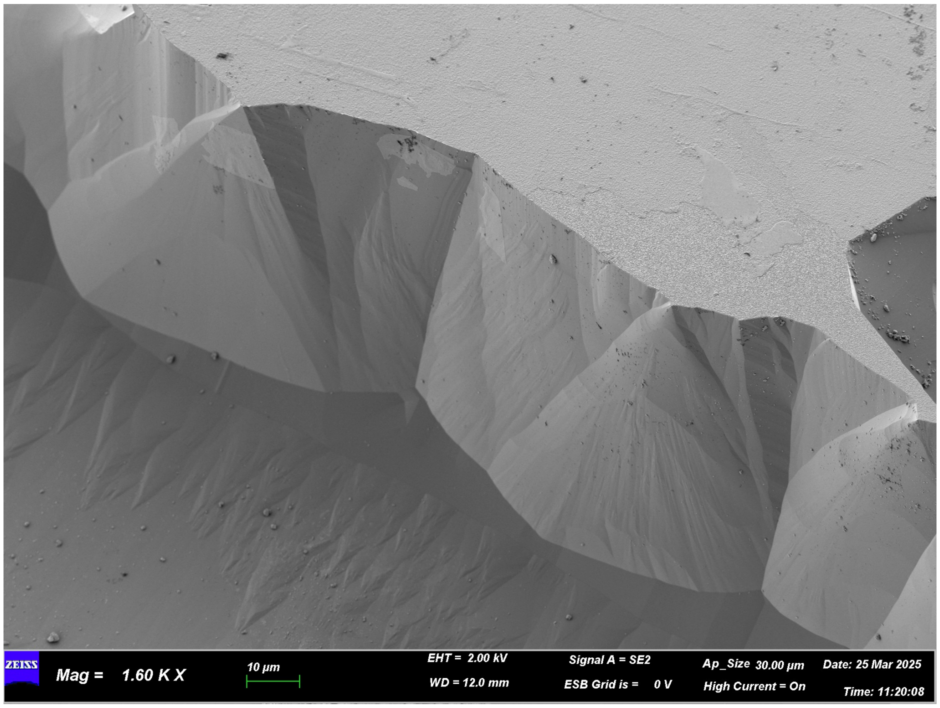

Figure S8. Sidewall of the aperture in the STO(110) sample. The sidewall corresponds to the $[1\bar{1}0]$ direction and its height is about 100 µm.
